# Individual Differences in the Impact of Distracting Environmental Sounds on the Performance of a Continuous Visual Task in Older Adults

**DOI:** 10.3390/brainsci14111048

**Published:** 2024-10-23

**Authors:** Leanne Richards, Neil Carter, Claire J. Hanley, Claire Barnes, Huw Summers, Alison Porter, Andrea Tales

**Affiliations:** 1 School of Medicine, Swansea University, Singleton Park, Swansea SA2 8PP, UK; 836713@swansea.ac.uk (L.R.); a.m.porter@swansea.ac.uk (A.P.); 2 Centre for Ageing and Dementia Research, Swansea University, Singleton Park, Swansea SA2 8PP, UK; 3 School of Psychology, Swansea University, Singleton Park, Swansea SA2 8PP, UK; n.carter@swansea.ac.uk (N.C.); c.j.hanley@swansea.ac.uk (C.J.H.); 4 Biomedical Engineering, Swansea University Bay Campus, Fabian Way, Swansea SA1 8EN, UK; c.m.barnes@swansea.ac.uk (C.B.); h.d.summers@swansea.ac.uk (H.S.)

**Keywords:** ageing, attention, auditory distraction

## Abstract

**Background/Objectives:** Vulnerability to sound distraction is commonly reported in older adults with dementia and tends to be associated with adverse impacts on daily activity. However, study outcome heterogeneity is increasingly evident, with preserved resistance to distraction also evident. Contributory factors may include individual differences in distractibility in older adulthood per se, and failure to consider the influence of how difficult a person found the test. **Methods:** We therefore measured distractibility in a group of older adults by comparing the performance of a primary visual task (Swansea Test of Attentional Control), which includes an adaptive algorithm to take into account how difficult a person finds the test under both no-sound and sound conditions. **Results:** Analysis revealed no significant difference in group mean performance between no-sound versus sound conditions [t (33) = 0.181, *p* = 0.858; Cohen’s effect size *d* = −0.028], but individual differences in performance both within and between sound and no-sound conditions were evident, indicating that for older adults, distracting sounds can be neutral, detrimental, or advantageous with respect to visual task performance. It was not possible to determine individual thresholds for whether sound versus no-sound conditions affected a person’s actual behaviour. **Conclusions:** Nevertheless, our findings indicate how variable such effects may be in older adults, which in turn may help to explain outcome heterogeneity in studies including people living with dementia. Furthermore, such within-group heterogeneity highlights the importance of considering a person’s individual performance in order to better understand their behaviour and initiate interventions as required.

## 1. Introduction

The unexpected and variable nature of our environment means individuals are constantly bombarded with sensory stimuli (e.g., touch, taste, smell, sound, vision). The brain’s information processing resources appear mediated (at least in part) via a flexible, responsive but threshold-driven attentional control system. This facilitates the processing of specific, behaviourally pertinent, multimodal information and inhibits, reduces, attenuates, or interferes with the processing of irrelevant but distracting stimuli, which, together with executive function, working memory, planning, metacognition, and goal-directive behaviour [1], maintains priority behaviour [2,3,4,5,6,7,8]. The functional integrity of various aspects of the attentional control system can be significantly impaired in older compared to younger adults, for example, an increased vulnerability to automatic attentional capture and distractibility by task-irrelevant stimuli [7,9,10,11,12], with disproportionate vulnerability to distraction typically reported in older adults with dementia. The automatic distracting effects of salient environmental sounds [13] form part of daily life and, beyond a certain threshold, can capture and reorient attention in order to process their origin in order to determine their relevance to current behaviour, their potential for danger, and, if necessary, to drive beneficial behavioural change. Too low a threshold for sound distraction (over-distractibility) can, however, be detrimental to behaviour and, in older adults, particularly those living with cognitive decline and dementia, may have an adverse impact on various aspects of daily life such as driving, work, task completion, concentration, environmental interaction, and social activities [12,14,15,16,17]. Over-distractibility can also detrimentally affect walking and gait in older adulthood, particularly in conjunction with dementia and cognitive impairment, increasing the risk of falls, hip fractures, disability, and immobility, and thus increased isolation, loneliness, and reduced independence [18,19,20,21,22,23,24,25,26].

Early detection of a propensity for over-distractibility, particularly in older individuals living with dementia, could ensure that the person living with it, and those close to them, together with the health and social care community, can be made aware of its occurrence, the situational triggers, and potential consequences, thus raising the potential for successful intervention.

The computer-based psychophysics technique typically used to study the functional integrity of resisting distraction in older adults requires the participant to perform a primary visual task in the presence and absence of auditory distraction. It is common for such studies to reveal poorer group mean attentional control or increased distractibility in terms of slowed reaction time (RT) and/or reduced accuracy under sound compared to no-sound conditions. Similarly, when focusing specifically on the role of multimodal information integration, processing, and modality, there is a modulation of behavioural performance [27]. What is of particular interest is the role modalities have when working individually or multimodally. For example, simultaneous multimodal input has been shown to increase early attentional processing of visual stimuli, cognitive effort, and conflict, whereas tactile visual input creates a larger impairment with multitasking, believed to be modulated by activity in the premotor cortex and visual areas [27]. This has been supported by additional research that shows modality pairing and compatibility influence response selection dual-task performance and interference effects [28,29]. Increasingly apparent, however, is the equivocal nature of such effects in older adults and older adults with dementia, with some results revealing preserved performance in the presence of auditory distraction [8,12,13,15,20,30,31,32,33,34,35,36,37,38,39,40,41].

Inter-individual variability factors may include differences in thresholds for, or functional integrity in, distractor detection, processing capability, suppression, speed, attentional capture, attenuation or blocking of the sensory processing of distracters, and the degree of high-level control available to command a specific level of engagement irrespective of causality. Furthermore, the typically inherent variation (e.g., high kurtosis/presence of outliers) in RT performance within groups may also be a contributory factor to such outcome variability. This can be related to individual differences in strategic processing [12,42], the functional integrity of other brain processes [43], motivation [8,39,40,41], demographic, and other methodological and morbidity-related factors, which may act independently, cumulatively, or interactively [12,22,43,44,45,46,47,48]. In addition, studies in this area have not tended to consider individual differences in task performance capability (e.g., the degree to which individuals find a given primary visual task difficult or easy and how long, or over how many trials, it takes a person to reach their maximum performance), which may further contribute to study outcome heterogeneity.

Interestingly, recent evidence suggests that education plays a pivotal role in mitigating the impact of distraction [49]. Therefore, enhancing opportunities for learning across the lifespan could enhance vigilance. Research suggests that the neural mechanisms underpinning individual differences in distractibility relate to the influence of education on frontal–parietal connections, particularly those within the right hemisphere. Additional research also postulates that regulation of the basal ganglia and thalamus via the prefrontal cortex is integral for successful sensory filtering [50]. Consequently, interventions designed to strengthen prefrontal connectivity could serve to bolster attentional control. For example, transcranial direct current stimulation (tDCS) to the dorsolateral prefrontal cortex (dlPFC) has previously been demonstrated to increase processing speed in relation to the STAC task [51,52] and, more recently, appears to be beneficial in relation to hazard perception [53]. Furthermore, such within-group heterogeneity highlights the importance of considering a person’s individual performance in order to better understand their behaviour and initiate intervention as required.

As with other aspects of brain function, irrespective of cause, such variation in group mean study outcome for older adults and older adults with dementia may be indicative of significant ‘normal’ individual variability in the extent to which any given salient sound detrimentally affects visual task performance. Knowing more about such individual differences in distractibility in older adults per se would better inform research and interventions for older adults living with dementia.

In order to highlight the potential for functional heterogeneity within such processing in cognitively healthy ageing, we present both group and individual data in the present study. We investigated the effect of real-life environmental sounds upon the performance of a difficult (high processing load) continuous visual attentional control task (the STAC test [Swansea Test of Attentional Control]) [47,54], compared to when no sounds were presented. As described in the Methodology section, the STAC is a continuous stimulus presentation paradigm that not only better represents real-life environmental situations, as opposed to delivering discrete trials in blocks that systematically vary task demands [55], but also takes into account a person’s own task performance ability.

## 2. Materials and Methods

### 2.1. Ethics

Ethics approval was granted by the Swansea University College of Human and Health Science (reference number LR_01_-11-22) and the study was conducted in accordance with the principles of the Declaration of Helsinki. Written informed consent was given by each participant, and all participants were debriefed after participation.

### 2.2. Participants

The number of participants, 34 [age range of 60 to 79 years; mean age = 68.03, SD = 5.48; 12 males, 22 females], was based on the performance of a two-tailed, repeated measures *t*-test, with a significance level of (α) = 0.05, an effect size (E) = 0.5, and power (ß) = 0.8, for comparison of STAC test performance under sound and no-sound conditions. Group mean MOCA [56] performance = 27.26 and standard deviation (SD) = 2.15. All participants were community-dwelling and fluent English speakers, with self-reported normal or corrected-to-normal vision and self-reported and tested hearing (using the RNID online hearing test [57] at the start of the testing session). Exclusion criteria included ongoing, or a history of, significant psychological, neurological, psychiatric conditions, or health conditions and poor mobility. None of the participants had consulted a general practitioner (GP) or memory services with concerns about their cognitive function. Medication could not be controlled but it was related to general ageing-related conditions (excluding dementia and cognitive decline) and stable, i.e., all participants had been taking their medication for some time. None of the participants reported unusually high exposure (at work or home/normal life) to any of the 30 different sounds presented under the ‘sound presentation’ condition of the STAC test.

### 2.3. STAC Test [Swansea Test of Attentional Control]

The STAC test [58] evokes selective attention, task monitoring, and response inhibition components of attentional control and thus simulates the natural and complex demands of continuous visual environmental monitoring and interaction within a single test, giving it high ecological validity. Moreover, the continuous nature of the STAC test better represents real-life environmental situations, unlike tasks that deliver discrete trials in blocks with varying task demands [55]. The STAC test includes an adaptive algorithm designed to track performance called Parameter Estimation by Sequential Testing (PEST) [59], which adjusts stimulus presentation speed on the basis of prior stimulus (target) response times (RT) in order to ensure that the task is performed in accordance with individual differences in how easy or difficult a person finds a test. STAC performance oscillates around an individual’s performance threshold, pushing them just out of range of their comfort zone before adjusting to a more manageable speed. The STAC test performance outcome/indicator is therefore the final speed of performance (measured in symbols per minute; ‘SPM’ performed by the end of the trial whilst able to maintain an accuracy rate of 75%). This avoids the potential outcome heterogeneity resulting from individuals’ capabilities, which may confound the effects of auditory distracters. The environmental sounds were not of relevance to the primary visual task. In addition, we displayed each participant’s results for the sound versus no-sound conditions of the STAC test in order to highlight potential individual differences.

The task was presented on a laptop with a 17.3″ screen and a resolution of 1920 × 1080 p; the viewing distance was approximately 520 mm. The task software automatically adjusts the overall area of the task such that the full vertical height of the screen is used, and the width is adjusted to be the same, thereby giving a square task area. Similarly, the symbols were also automatically sized to be square, occupying the same proportion of the task area, regardless of the screen’s aspect ratio and resolution. On the laptop used for this experiment, the symbols measured 36.2 mm on each side, yielding a visual angle of 3°59′.

**Figure 1:**
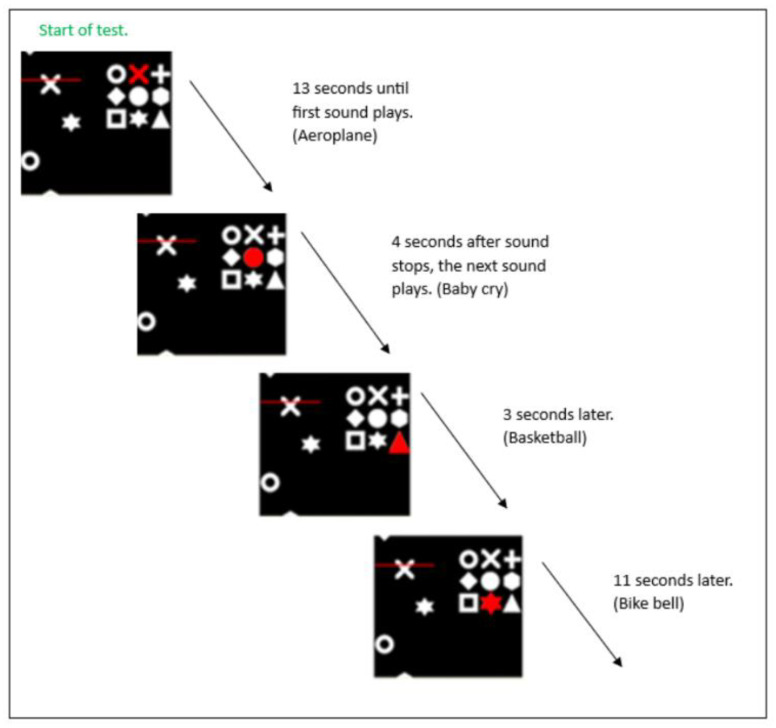
The STAC test. A target is identified within the 3 × 3 matrix of symbols (right); for both no-sound and sound conditions [58].

The task requirement is to identify the target (red symbol) within the 3 × 3 matrix of symbols and search for matching symbols amongst an array of three tracks of symbols (on the left) (see Figure 1). When a matching symbol appeared amongst the three tracks of the search array, which scrolled up the left-hand side of the screen, participants were required to press the keyboard spacebar as the symbol crossed behind the red line (as opposed to before or after). The matrix target changed at regular intervals throughout the task such that participants had to remain vigilant in order to consistently update their search criteria, while simultaneously monitoring the tracks to identify matching items and ignore distractor symbols. The target changed every 10 seconds (s) but was delayed if the current target was already present in the tracks (e.g., the target did not change to a different symbol if the current target was moving up the tracks). In such instances, the corresponding delay was added to the total run time (initially set to 390 s, resulting in approximately 9 target changes for all participants). Performance (measured in ‘symbols per minute per column’ (abbreviated to SPM) was adjusted to maintain accuracy around a commonly adopted 75% correct criterion, using the PEST algorithm [59]. The task began at 41 SPM. After a minimum of 4 target changes (for the first block), SPM was calibrated on the basis of performance accuracy, increasing or decreasing with a step size between 20 and 60 SPM. The participants’ thresholds are the average SPM at which the task was performed across the duration of the task. Participants were asked to try hard to keep up with the symbols and not to be discouraged if they missed some of the targets because the task was designed to be difficult. Participants were informed that the symbols would move up the screen at different speeds during the test. They were also told that they would receive no performance-related feedback over the duration of the test.

The environmental sound clips used in this study (N = 30, see Table 1) were taken from a list of typical UK-based environmental sounds previously determined by Richards et al. [60] (and see also Marcell et al. [61]), in preparation to be universally perceived by the study participants as both highly distracting and unpleasant. The sound clips used in this study were played in a fixed order to minimise extraneous variables. To avoid anticipation of environmental sound, thus potentially affecting attention control, the onset of each sound was played at random intervals. For example, the first environmental sound clip was played 13 s after the start of the test, the next sound 4 s later, then 3 s later, followed by a further sound 11 s later, and so forth [see Figure 1]. Each environmental sound clip (at 80 dB) lasted approximately 3 s, with no temporal overlap between them. A static background sound clip of a bird song was played for the full duration of the sound condition (at 25 dB, equivalent to a quiet whisper) to simulate a continuous noise environment.

### 2.4. Procedure

Participants were seated in a quiet dedicated research lab at Swansea University and instructed how to perform the STAC test using verbal, written, and illustrated instructions and were provided with the opportunity to ask questions. Participants were then seated comfortably in front of the monitor of the HP Envy laptop with NVIDIA graphics processing unit, featuring accelerated output to the device display. All participants performed a practise trial of the ‘no-sound’ condition (of approximately 3.2 min duration), to ensure that they were able to see the stimuli, understand the task requirements, and physically perform the task (approximately 3.2 min in duration). The main STAC test was then presented twice, once for the sound condition and one for the no-sound condition (counter-balanced), with each presentation lasting approximately 6 min and 48 s, with approximately 4 min in between (to allow any visual disturbances, such as side effects of the symbol movement) to dissipate. In order to maximise their potential for distraction, participants were not informed prior to the start of testing that they would be subjected to a range of environmental sounds under one of the testing conditions. The 30 different environmental sounds were presented throughout the sound condition of the task at pre-specified times (i.e., after the first sound clip, the sounds were presented at 2, 5, 6, and 9 s, taking into account the duration of the sounds themselves). Once all 30 sounds had been presented, the list was repeated in the same order until the test ended. Upon task completion, the participants were debriefed as to the nature of the study and offered the opportunity to have any questions answered.

## 3. Data Analysis and Results

The performance measures for the sound vs. no-sound condition comprised the number of symbols responded to per minute by the end of the study (SPM) whilst able to maintain an accuracy rate of 75%.

**Figure 2:**
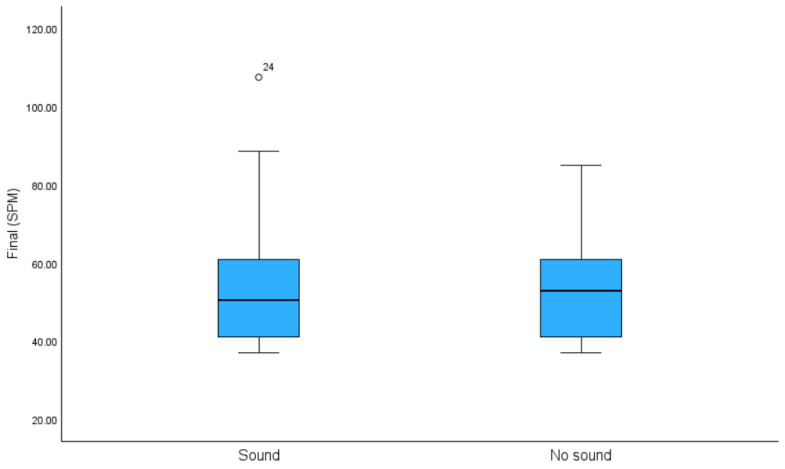
Box plot of the group mean final SPM for the STAC under both the sound and no-sound conditions.

**Figure 3:**
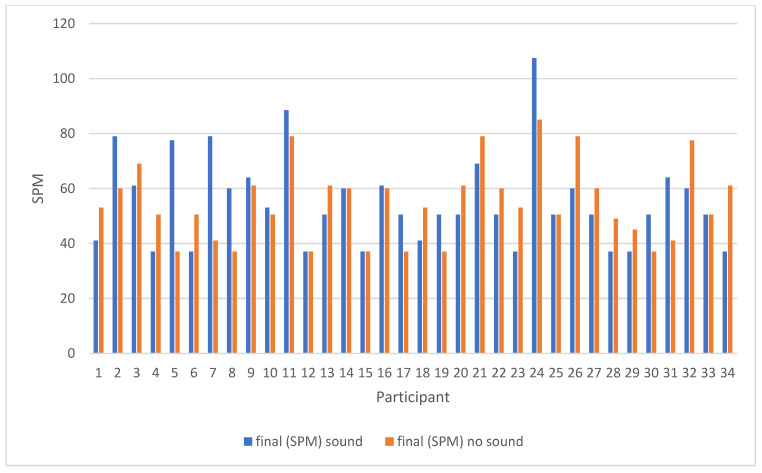
The final SPM scores for the sound and no-sound performance for all the individual 34 participants.

Although the sound condition increased the mean final SPM by 0.5 symbols per minute (i.e., improved performance) compared to the no-sound condition, a repeated measures *t*-test revealed that this difference failed to reach statistical significance [t (33) = 0.181, *p* = 0.858; Cohen’s effect size *d* = −0.028]. See Figure 2 and Figure 3.

Although there is some indication that, for males, sound improves performance (SPM) compared to no sound, this did not reach statistical significance. ANOVA revealed no significant main effect of sex [F (1, 32) = 1.082, *p* = 0.306], no significant main effect of condition (sound versus no sound) [F (1, 32) = 0.157, *p* = 0.695], and no significant sex by condition interaction [F (1, 32) = 0.582, *p* = 0.451]. Note, however, that this is a post-priori analysis and the study was not powered to formally examine the effects of sex upon performance.

Despite the fact that the order of the sound and no-sound condition presentation order was counter-balanced, it is possible that whether the sound or no-sound condition was present first may have affected the results. When the sound condition was presented first, SPM performance was better in the ‘no-sound’ condition compared to the ‘sound’ condition. Conversely, when the no-sound condition was presented first, SPM performance was better in the ‘sound’ compared to the ‘no-sound’ condition. Statistical analysis with ANOVA (main effect of order, main effect of condition), however, revealed that these effects were not significant (Table 2). There was no significant main effect of the sound [F (1, 32) = 0.008, *p* = 0.930], no significant main effect of task order [F (1, 32) = 1.419, *p* = 0.242], and no significant sound condition by task order interaction [F (1, 32) = 0.587, *p* = 0.449].

## 4. Discussion

The aim of this study was to examine the nature of distractibility in older adults with particular emphasis on individual differences. Although the sound condition increased the group mean SPM and thus STAC performance by 0.5 symbols per minute (Table 1 and Figure 2), this effect failed to reach statistical significance [*p* = 0.86, Cohen’s effect size *d* = 0.028]. Typically, such a result would be reported as indicative of resilience to distraction in older adulthood, i.e., that the distracting environmental sounds did not detrimentally affect their performance of a difficult visual task.

However, presenting the results for each individual within the group (Figure 3 and Appendix B) reveals a wide range of performance (SPM), not only for both the sound and the no-sound conditions, from 37 to 107.5 and 37 to 85, respectively, but also with respect to the difference in SPM performance between these conditions (no-sound SPM–sound SPM), which ranged from −40.5 to 24 (Table 3). Although it was not possible to determine from this study how the sounds under laboratory conditions translate to physiological responsiveness or real-life behaviour, it is clear that for some individuals, distracting environmental sound had no effect on the performance of the STAC, whereas, for others, it was either detrimental (i.e., reduced performance) or advantageous (increased performance). Although we were not able to determine what degree of difference is meaningful in real-life behaviour, it is clear that high variation can exist in response to the same sound and no-sound conditions, and with respect to the difference in performance between these two conditions. This may be a contributory factor to study outcome variability in this research area, depending upon the mix of ‘responders’ within different studies. The results also highlight the importance of investigating the performance of the individuals themselves with respect to real-life and clinical relevance, especially in terms of determining personal triggers for or resilience to distractibility. These results in older adults also emphasise how difficult it may be to determine or predict how the performance of someone with mild cognitive impairment (MCI) or dementia may differ from healthy ageing and that one probably cannot predict performance based on diagnosis per se, but would rather have to adopt a more personalised, possibly situational-specific approach to testing and intervention.

Future studies using the PEST paradigm may be able to determine how functions such as distractibility change over time in relation to what is the normal performance of an individual, with particular relevance to individuals at risk of transitioning from SCD or MCI to dementia. Similarly, further practical/real-life investigations may help to identify individuals whose behaviour may be likely to be detrimentally affected by salient, distracting environmental sounds. Further research is required in order to determine factors such as the familiarity and relevance of distracting sounds [8,13]. Although the distracting environmental sounds were, in principle, irrelevant to the visual task, it is possible that participants assigned varying relevance to some of them, which may have influenced performance [13]. Additional research is required to determine the causal mechanisms of individual differences in distractibility. In addition, there is some evidence that distractors have less effect when the processing load of the primary task is high. There may be individual differences related to a scenario where the distractor is detected but the attentional response to it is suppressed, or that the sensory processing of distractors is attenuated or blocked under high perceptual load, or that distractors are detectable even under high task engagement but that stronger task engagement circumvents the actual switch of attention to the distracting stimulus [8].

There is a need for further exploration as to the potential reasons behind individual differences and the non-significant results found. Possible areas for exploration would benefit from research addressing the impact of auditory distraction on task performance in older adults via multimodal processing and regulation of sensory processing. Furthermore, it may be beneficial to explore the sensory modalities singularly and as multimodal, given that research has shown that how sensory stimuli are presented influences task performance. The practical implications of understanding individual differences in distractibility will aid future interventions in cognitive enhancement (e.g., tDCS), attention deficit hyperactive disorder management, and cognitive training measures such as auditory cognitive training to improve perception and regulation.

In order to reduce potential confounding effects in the present study, the STAC included an adaptive algorithm designed to track performance. PEST [59] adjusts stimulus presentation speed on the basis of prior stimulus (target) response times in order to ensure that the task is performed in accordance with how easy or difficult a person finds a test. In addition, despite counterbalancing, the data were also analysed with respect to potential order effects, namely whether the sound or no-sound condition was presented first, revealing no significant effects. This, together with previous research utilising STAC, which has demonstrated stable performance over four repeat exposures to the task [54], attests to the robust nature of the STAC test.

### Potential Limitations and Future Studies

Potential limitations include the fact that we did not measure performance over time and that we did not include parallel physiological testing that would have enabled us to determine if there was an initial startle effect followed by habituation and, if so, whether individuals varied significantly in this pattern of response and whether any sounds had a greater effect than others. Future studies will employ these physiological measures to assess the degree to which an individual’s attention maintenance and performance correlate with physiological changes associated with environmental sound. Finally, while we believe the stimuli do reflect real-world demands, they could be refined in future studies. Future studies will aim to add more complex sounds, involving multiple types of sounds that may co-occur or overlap. Furthermore, it would be beneficial to re-design the study to focus on more dynamic environments that take advantage of previous research addressing selective attention (e.g., the cocktail party effect). This may include, but is not limited to, running this study in a library, coffee shop, or cafeteria. The benefit of introducing more dynamic environments would provide greater complexity of sounds that are heard (e.g., multi-talker situations, the participant’s name being called, shouting, and increased risk of unexpected noise and temporal overlapping of auditory stimuli), that, in turn, would increase the ecological validity and reliability of results, whilst supporting the development of auditory distraction interventions in an individualised manner, for older adults and individuals with MCI and dementia.

## 5. Conclusions

The findings of this paper indicate how variable the impact of sound is in older adults, which in turn may help to explain outcome heterogeneity in studies including people living with dementia. Furthermore, such within-group heterogeneity highlights the importance of considering a person’s individual performance in order to better understand their behaviour and facilitate interventions as required.

## Figures and Tables

**Table 1:** Group mean final SPM (SPM), standard deviation (SD), and 95% confidence intervals (CI) for both the sound and the no-sound conditions.

No-sound condition	Range of SPM = 37 to 85
	Median SPM = 53
	Mean SPM = 54.68
	SD = 18.83
	95% CI = 54.68 ± 4.719
Sound condition	Range of SPM = 37 to 107.5
	Median SPM = 50.5
	Mean SPM 55.18
	SD = 16.48
	95% CI = 55.18 ± 5.623

**Table 2:** Potential order effects of final SPM with respect to whether the sound or no-sound condition was presented first.

		Mean Final (SPM)	Standard Deviation (SD) of Final SPM	Median Final SPM
Sound condition presented first	Final SPM sound condition	50.97	12.19	50.5
	Final SPM no sound	52.87	14.43	53
	Difference = (sound–no sound)	−1.9		
No-sound condition presented first	Final SPM sound condition	58.5	19.26	53
	Final SPM no-sound condition	56.11	13.95	53
	Difference = (sound–no sound)	2.39		

**Table 3:** Potential sex-related differences in SPM under the sound and no-sound STAC conditions.

	Mean Final SPM for the Sound Condition	Mean Final SPM for the No-Sound Condition
male	59.79	56.42
female	52.66	53.73

## Data Availability

The original contributions presented in the study are included in the article, further inquiries can be directed to the corresponding author.

## Appendix A

**Table A1:** Sounds used in STAC test.

Airplane	Blowing nose	Clear throat	Gargling	Mosquito	Snoring
Baby cry	Burp	Cymbals	Glass breaking	Motorcycle	Telephone
Basketball	Car crash	Dog bark	Hammering	Police siren	Trumpet
Bike bell	Car horn	Doorbell	Jack hammer	Scream	Water drip
Blinds	Child cough	Drill	Lawnmower	Sneeze	Zipper

## Appendix B

**Table A2:** The performance (SPM) under sound and no-sound conditions and the difference in performance between the two conditions for each participant.

Participant	SPM Sound Condition	SPM No-Sound Condition	SPM Difference (No Sound–Sound)
1	41	53	12
2	79	60	−19
3	61	69	8
4	37	50.5	13.5
5	77.5	37	−40.5
6	37	50.5	13.5
7	79	41	−38
8	60	37	−23
9	64	61	−3
10	53	50.5	−2.5
11	88.5	79	−9.5
12	37	37	0
13	50.5	61	10.5
14	60	60	0
15	37	37	0
16	61	60	−1
17	50.5	37	−13.5
18	41	53	12
19	50.5	37	−13.5
20	50.5	61	10.5
21	69	79	10
22	50.5	60	9.5
23	37	53	16
24	107.5	85	−22.5
25	50.5	50.5	0
26	60	79	19
27	50.5	60	9.5
28	37	49	12
29	37	45	8
30	50.5	37	−13.5
31	64	41	−23
32	60	77.5	17.5
33	50.5	50.5	0
34	37	61	24
